# Biobehavioral approach to distinguishing panic symptoms from medical illness

**DOI:** 10.3389/fpsyt.2024.1296569

**Published:** 2024-05-08

**Authors:** Natalie C. Tunnell, Sarah E. Corner, Andres D. Roque, Juliet L. Kroll, Thomas Ritz, Alicia E. Meuret

**Affiliations:** ^1^Department of Psychology, Southern Methodist University, Dallas, TX, United States; ^2^Department of Psychiatry & Behavioral Sciences, The University of Kansas Medical Center, Kansas City, KS, United States; ^3^Primary Care Department, Miami VA Healthcare System, Miami, FL, United States; ^4^Department of Palliative, Rehabilitation and Integrative Medicine, The University of Texas MD Anderson Cancer Center, Houston, TX, United States

**Keywords:** panic disorder, somatic symptoms, medical comorbidities, differential diagnosis, dyspnea, long covid, biobehavioral health

## Abstract

Panic disorder is a common psychiatric diagnosis characterized by acute, distressing somatic symptoms that mimic medically-relevant symptoms. As a result, individuals with panic disorder overutilize personal and healthcare resources in an attempt to diagnose and treat physical symptoms that are often medically benign. A biobehavioral perspective on these symptoms is needed that integrates psychological and medical knowledge to avoid costly treatments and prolonged suffering. This narrative review examines six common somatic symptoms of panic attacks (non-cardiac chest pain, palpitations, dyspnea, dizziness, abdominal distress, and paresthesia), identified in the literature as the most severe, prevalent, or critical for differential diagnosis in somatic illness, including long COVID. We review somatic illnesses that are commonly comorbid or produce panic-like symptoms, their relevant risk factors, characteristics that assist in distinguishing them from panic, and treatment approaches that are typical for these conditions. Additionally, this review discusses key factors, including cultural considerations, to assist healthcare professionals in differentiating benign from medically relevant symptoms in panic sufferers.

## Introduction

Panic disorder (PD) is characterized by panic attacks (PAs), distinct and unexpected episodes of intense somatic and cognitive symptoms that peak within minutes. Benign somatic symptoms experienced during PAs (i.e., palpitations, dyspnea, chest pain, dizziness) often resemble more malignant symptoms of medical illness such as arrhythmias, hypertension, or asthma, among others. However, these somatic symptoms are non-specific and can occur in other psychosomatic illnesses, such as somatoform disorder, or in the absence of disease entirely (1). This can lead to considerable uncertainty among biobehavioral researchers and healthcare professionals about the diagnosis and treatment of patients. The DSM-5 diagnostic criteria require the presence of a minimum of four symptoms to meet the threshold for Criteria A for PD (2). Multidimensional characteristics such as levels of alexithymia, interoceptive accuracy, age, comorbidity with other several psychiatric disorders, sex, and medical history can further complicate diagnosis (3–5). Thus, panic patients frequently have catastrophic beliefs about the origin of these somatic symptoms (6). Unsurprisingly, panic sufferers spend considerable effort seeking diagnoses and treatment for their symptoms.

Although the lifetime prevalence of PD (3.7%) is lower than other anxiety disorders, PAs are frequent (22.7%), and the disorder results in disproportionately high healthcare utilization, particularly in acute care settings (7). Upwards of 12% of all patients presenting to the emergency department (ER) meet the criteria for PD; when chest pain is the presenting complaint, the percentage increases to 20-35% (8). Patients often reject reassurance of the benign nature of symptoms; for example, most individuals who present to the ER with chest pain seek continued medical treatment even after a cardiac diagnosis is ruled out (9, 10). Follow-up diagnostic assessments such as angiograms are expensive and burdensome for patients and providers (11, 12). Misdiagnoses and diagnostic assessments are costly and contribute to delayed treatment, with the duration of treatment delay PD ranging from 9 years to 15 years (13, 14). Left untreated, PD can result in numerous negative consequences, including disability, financial stress, reduced ability to work, and increased rates of mortality (15–17). These consequences are exacerbated in medical populations experiencing complications from their existing conditions and can maintain poor health behaviors (18). For instance, cardiopulmonary patients have higher anxiety levels and fears about exercise, which contributes to avoiding exercise essential to their health (18). Vice versa, a patient’s symptom misinterpretation or denial or a healthcare provider’s misdiagnosis can lead to a critical treatment delay for acute or chronic somatic conditions (19–21).

Given the high number of medical reassurance-seeking panic patients, research has examined their ability to differentiate between benign and malignant symptoms. Findings suggest vast differences in discriminative detection and report somatic distress. Contributing factors include personality traits, alexithymia, history of abuse, medical illnesses, comorbid psychiatric or medical conditions, and lack of proper explanations or therapeutic care (3, 21–23). Elevated somatization, in which psychological distress manifests as bodily symptoms (24), is also a factor. It is important to note that diagnostic nomenclature (2) prohibits a diagnosis of PD if the origin of panic symptoms is a direct result of a medical condition, such as dyspnea from asthma. However, since panic symptoms mimic several critical medical conditions, the differential diagnosis is far from straightforward. For example, paresthesia, a less frequent yet significant indicator of severe PA, is also a common symptom of epilepsy (25, 26), hyperventilation-accompanied asthma (27, 28), and non-epileptic seizures, and is a reported side effect of medications, such as topiramate (29). Unsurprisingly, PAs are the most common psychiatric differential diagnosis for seizure disorders (30).

Misattributing panic as a medically-relevant condition likely leads to underestimating PD in medical populations and significant management problems in primary and acute care settings. Thus, reliably differentiating benign from malignant somatic symptoms in PD is vital. At a minimum, guidance should enable scientists and healthcare professionals to recognize psychiatric symptoms to ensure proper therapeutic attention. Bodily sensations are complex and heterogeneous, and attempts to assign “typical” versus “non-typical” somatic symptom presentation to medical or psychiatric diagnoses are unlikely fruitful. A more valid approach might incorporate symptom frequency, duration, and antecedents in the context of other differentiating factors to guide therapeutic action.

The aim of this review is to report the current literature on somatic panic symptoms and discuss common differential diagnoses. We describe the selected symptoms, their prevalence in general populations and within PD, symptom causes, recommendations for distinguishing benign from potentially malignant symptoms, and symptom treatment. Notably, attention and coverage in the literature across symptoms varies considerably, resulting in differing degrees of detailedness. This review was conceived as a resource for biobehavioral researchers and healthcare professionals who study or treat patients with symptoms compatible with PD and, as such, do not provide an in-depth conceptual analysis on the interplay of symptoms in psychiatric and somatic illnesses (e.g., 6, for conceptual review).

## Methods

As a first step, we identified in the literature six relevant symptom domains of panic and somatic illness that complicate diagnostic differentiation or co-occur with elevated rates: palpitations, shortness of breath, chest pain, nausea, dizziness, and paresthesias. The selected symptoms are the most frequently reported and often the most distressing to patients (8, 31), and most prominently pose a challenge for integrative healthcare professionals when attempting to distinguish benign from medically relevant symptoms. Searches were performed in databases PubMed, Cochrane Library, and PsycINFO up to February 2, 2024. Relevant papers were identified using a combination of both free text and MeSH terms for panic disorder, the individual symptom domains, associated medical conditions, differential diagnosis, and treatment/therapy (e.g., when searching for panic, shortness of breath/dyspnea and asthma, we used the following syntax: ((panic disorder[MeSH Terms]) AND (asthma[MeSH Terms]) AND ((dyspnea[MeSH Terms]) OR (breathlessness[Text Word]) OR shortness of breath [Text Word])) AND (differential[Text Word]) AND (diagnosis[Text Word])). Because these searches typically yielded fewer papers, we also searched reference lists of identified papers, textbook chapters, and handbooks we have archived over the years. We excluded older papers from domains that had more recent, up-to-date publications. In identifying relevant somatic illness in this review, we first referenced existing empirical evidence and educational materials for medical professionals (e.g., textbooks) that specify the most frequent diagnostic considerations for the symptoms. We chose somatic illnesses that are characterized, in part, by one of the panic symptoms and focused on those that are most frequently conflated or comorbid with panic.

## Palpitations

### Prevalence and presentation

Patients experience palpitations as a missed/irregular heartbeat (ectopic beat or extrasystole) or racing/pounding heartbeat (awareness of sinus rhythm and/or stronger contractile force of the heart muscle). Of the 13 PD DSM-5 criteria, heart palpitations are the most prominent and widely reported physical symptom and are commonly cited as the most severe (6, 31, 32). They are one of the most common reasons for cardiologist referrals (33, 34), with one study indicating that 16% of primary care outpatients report palpitations (35), and have a community prevalence of 6-11% (36). Notably, the medical literature lists over 30 different cardiac and non-cardiac causes and triggers of palpitations (37). Patients reporting benign palpitations experience negative outcomes over time, such as decreased quality of life, increased psychiatric morbidity, increased disability, and persistent concern over heart health (38).

### Differential diagnosis

#### Medical event versus panic attack

Palpitations may be related to arrhythmias or other coronary abnormalities. Arrhythmias occur when a heartbeat is too quick, too slow, or has an irregular rhythm and can indicate more serious medical conditions, including heart failure, autoimmune disease, and liver failure (39). Most cases of palpitations are unrelated to significant clinical arrhythmias or abnormal heartbeats. Indeed, most patients with arrhythmias do not notice or express concern over palpitations (40). Current guidelines recommend that all patients presenting with a chief complaint of palpitations undergo a physical examination, electrocardiogram (ECG), and detailed history. The latter is critical for identifying symptom origin. Holter monitors should play a limited role in evaluating patients with palpitation symptoms and should only be used if symptoms occur reliably every 24 hours (36).

Palpitations are among the most common complaints in patients with mitral valve prolapse (MVP), which is a cardiac condition defined by the abnormal placement of the mitral valve leaflets during systole (41). In most cases, MVP is not life-threatening and does not require treatment (42), though some patients develop autonomic symptoms, including exercise intolerance, orthostasis, and syncope (43). The prevalence of MVP within the general population ranges from 2-3% and is typically diagnosed by using a stethoscope or 2-dimensional echocardiography to monitor for clicks or murmurs (42). Though often asymptomatic, palpitations can be accompanied by chest pain and dyspnea (41). A 2019 meta-analysis of 14 studies observed that panic patients were twice as likely to suffer from MVP than controls (44). Researchers have also identified a non-random aggregation of MVP in some family members of PD patients that may suggest a common genetic mechanism, namely a strong peak on chromosome 13q (e.g., 45). However, the literature remains equivocal, with other studies showing little evidence for an association between MVP and PD after correcting prior shortcomings (44, 46).

#### Associated characteristics

Previous research has utilized ECGs to determine patients with clinically significant arrhythmias and benign palpitations (1, 47). Strikingly, one study found that, compared to patients with benign palpitations, patients whose reports of palpitations coincided with actual arrhythmias had lower levels of somatization, hypochondriacal symptoms, and psychiatric morbidity, and none had PD (48). In contrast, a study examining the characteristics of patients with benign palpitations found that 39% of patients had a psychiatric disorder of any type, and 14% had PD (49). The same can be applied to resting heart rate (HR) perception. Compared to controls, patients with palpitations detect their HR less accurately in heartbeat interoception tasks (50–52). By contrast, panic patients tended to report arrhythmic HR without actual arrhythmias (50), thus indicating that the perception may be a result of top-down processes (53).

These findings also support the notion of palpitations being a highly inaccurate marker of arrhythmias, with little association between the severity of arrhythmias and cardio perception. Basic interoception research points to a potentially more decisive role of sympathetically mediated heart muscle contractility than the parasympathetically dominated sinus rhythm in the perception of cardiac activity (54). Additional psychological factors were illustrated in a study by Karsdorp et al. (55): Patients with chronic heart disease (CHD) and comorbid high trait anxiety reported more pronounced cardiac sensations to false HR feedback than patients with non-comorbid CHD. In contrast, during a false heartbeat detection task interspersed with true feedback, highly anxious individuals with a history of PAs accurately identified the same amount of true HR feedback trials as low-anxiety controls (56). Thus, perceptual bias due to a history of CHD combined with high trait anxiety could be a more likely moderator than somatic status. While adaptive in true alarm situations, over perception likely enhances morbid behaviors (e.g., avoidance of physical activity, overuse of medical resources) (57, 58). Comorbid anxiety was also related to worsened general health status and atrial fibrillation symptom severity in patients with atrial fibrillations (59).

Furthermore, benign palpitations are associated with being female, a sedentary lifestyle, and poor interoceptive sensitivity (51). Fear and anxiety surrounding exercise and the symptoms it might elicit are frequent in patients undergoing cardiovascular rehabilitation and have been associated with a more unfavorable clinical presentation at admission, including lower perceived mental health status (18). Healthcare providers overly concerned about patients’ fears (as visible by e.g., frequently checking heart rate during exercise) may inadvertently reinforce patients’ feelings, resulting in catastrophizing and avoidance of exercise, with adverse consequences for general health. Higher anxiety sensitivity in these patients is also associated with avoidance of exercise (60).

### Treatment

Medical treatment for benign palpitations usually provides little reassurance or symptom relief, leaving patients dissatisfied with care (61). Psychological treatments for palpitations are emerging, with illness and symptom perception acting as important treatment outcomes (62). Cognitive behavior therapy (CBT) has demonstrated effectiveness in reducing illness perception in patients with benign palpitations (38). Mindfulness-based stress reduction, an intervention that focuses on acquiring non-judgmental awareness and meditation techniques, has also yielded positive results (63). Techniques to increase relaxation, including meditation techniques, may also be a viable treatment for individuals with benign palpitations. A two-patient case study aimed at evaluating the efficacy of Prānāyāma (yogic) breathing and progressive muscle relaxation on the frequency of benign palpitations found that after two months with the techniques, patients experienced fewer episodes of palpitations and could relieve palpitations by practicing yogic breathing (64). Currently, no psychological treatment is widely used to aid in the reduction of MVP-related symptomatology. Some studies have observed the efficacy of aerobic exercise training and biofeedback on MVP, although these treatments have not been disseminated into routine medical and psychological practice (65).

## Shortness of breath/dyspnea

### Prevalence and presentation

Shortness of breath (SOB) is a symptom typically subsumed under the umbrella of dyspnea, encompassing the uncomfortable feeling of not having enough air, the urge to breathe, feelings of difficulty breathing, breathlessness, or air hunger (66). SOB is associated with an overall poorer quality of life (67). Several psychosocial variables have been proposed to elucidate the relationship between dyspnea and perceived well-being (68). In psychiatric settings, SOB is common, experienced in up to an estimated 87% of reported PAs, and is often rated among the most severe symptoms experienced during panic (31, 69). SOB is also a symptom in individuals presenting with agoraphobia, an anxiety disorder closely related to panic. SOB is a common symptom presentation in medical settings, both in emergency medicine and primary care. Up to 25% of patients entering the emergency department present with a primary complaint of dyspnea or SOB, making it the 8th most frequently reported principal reason for visit (70).

While the etiological mechanism of SOB in PD is unknown, some researchers postulate that dyspnea results from hyperventilation (71) or a false suffocation alarm (72). Ley (73) proposes that “severe fear experienced during hyperventilatory panic attack” results from intense dyspnea and perceiving little control over the experienced SOB. The suffocation false alarm theory proposes that spontaneous PAs result from a brainstem chemosensory system that is hypersensitive to rising CO2 levels, thereby triggering hyperventilation as a compensatory response and sensations of dyspnea (72). Despite its evolutionary salience, signaling the potential for loss of life, paradoxically, the experience of SOB is infrequently related to impending suffocation or lack of adequate oxygen saturation.

### Differential diagnosis

#### Medical event versus panic attack

Given high intra-individual variability in the perception of dyspnea across studies, differentiation of symptoms (e.g., “I felt like I could not get enough air”) as malignant or benign is quite challenging. There are key elements that distinguish between a medical event and a PA, with hallmark features of a PA as 1) duration of dyspnea being approximately 10 min, and 2) onset of SOB/dyspnea not predicted by any acute pulmonary or cardiovascular factors (2). Hallmark features of a medical event specific to the disease type are outlined below. However, generally, if there are indications from lung function tests or if the symptoms respond to medically appropriate medication in lieu of anxiolytics, a medical event rather than a PA is more likely the cause of symptoms. Regardless of psychological disorder or somatic disease state, the perception of symptoms likely varies on dimensions of intensity and affect (74, 75). Diagnosis for dyspnea often necessitates careful and detailed history taking, physical exam, and laboratory examinations, including chest film, ECG, and hematocrit. Most medical recommendations for dyspnea classification focus on distinguishing between cardiogenic or respiratory origin (76).

Acute SOB presents with a broad range of medical diagnoses, including chronic and life-threatening conditions. Approximately 85% of cases initially presenting with dyspnea are diagnosed with pulmonary or cardiovascular diseases (e.g., asthma, congestive heart failure, chronic obstructive pulmonary disease [COPD], pneumonia, pulmonary embolism, cardiac ischemia; 77). However, the experience of SOB outside of a medical event is typically unrelated to oxygen desaturation, and even in laboratory settings that induce dyspnea symptoms, oxygen saturation levels are remarkably stable, excluding at least some of these conditions (78).

In patients with COPD, a disease characterized by progressive and irreversible airflow obstruction, often due to a smoking history or prolonged exposure to air pollutants, SOB is frequent and medically relevant (79). For patients with a known diagnosis of COPD, taking a thorough examination of symptom duration is particularly necessary, as panic symptoms are characterized by acute onset (e.g., within minutes) and last for approximately ten minutes (2). In contrast, COPD exacerbations have a gradual onset and persistence, often lasting hours or days. Utilizing established medical indicators relevant to COPD disease processes (e.g., airway inflammation, gas exchange abnormalities; 79) can confirm if symptoms are more likely related to the underlying disease exacerbation than a panic. In more severe cases of COPD, dyspnea is elicited already by minor physical activities or can be present even during rest.

In patients presenting for pulmonary rehabilitation, fear of exercise and associated breathing difficulties have been associated with lower lung function and physical quality of life (18). PD is also highly prevalent in patients with COPD, with lifetime prevalence estimated to be 47% greater in patients with PD than in the general population (80). When patients with COPD also present with a PA, symptoms of the PA will be consistent with acute onset and SOB symptom elevation in a duration of minutes.

SOB is also a cardinal signal of asthma exacerbation (81). One study found that of the patients presenting to the ER with a primary complaint of SOB, 12.7% of patients were diagnosed with asthma (82). Studies have determined an elevated prevalence of anxiety disorders in patients with asthma (for reviews, see 6, 83), with an estimated comorbidity rate of asthma and PD between 6.5% and 24% in adults. One explanation of the high and varied comorbidity is the bi-directional relationship between panic and asthma symptoms. Hyperventilation, a commonly observed symptom in PD, leads to bronchoconstriction (84), a characteristic pathophysiological feature of asthma. Similarly, intense fear, panic, and other emotions can worsen bronchoconstriction (85) and elevate airway inflammation (86), thus generating sensations of dyspnea (74). Conversely, asthma exacerbations lead to symptoms greatly feared by PD patients, particularly dyspnea, and can enhance the perceived unpleasantness of such sensations (74, 75), thus increasing the frequency and intensity of PAs.

Asthma can be distinguished from the experience of panic by pulmonary function tests such as spirometry, reversibility testing with a bronchodilator, methacholine challenge tests to determine airway hyperreactivity, exhaled nitric oxide levels, and identifying antecedents to symptoms exacerbation (e.g., exposure to outdoor air pollution, food allergy, viral respiratory infections; 81). However, given that asthma pathophysiology is variable over time, some tests may not detect abnormalities indicative of asthma during symptom-free periods. Additionally, because asthma and PD are highly comorbid, it is essential to remember that patients presenting with SOB may meet diagnostic criteria for both disorders. Similar to other respiratory conditions, honing in on the duration of dyspnea (i.e., persistence for longer than 10 minutes typical of a PA) and if the dyspnea is associated with specific trigger(s), which are common to asthma exacerbations such as respiratory infections, air pollutants, allergens, exercise, or stress (81, 87), is important to distinguish if the dyspnea symptoms are more likely to be associated with disease course or a co-morbid panic attack.

While highly prevalent in respiratory disease, dyspnea can also indicate cardiac abnormalities. Cardiac disease accounts for 14.9% of SOB complaints in ED settings, with acute heart failure being a leading cause of hospitalization, morbidity, and mortality in patients presenting with dyspnea (82). Dyspnea is present in 56% to 86% of heart failure cases, and up to 86% of patients with cardiac disease have sought urgent care for breathlessness (88, 89). Dyspnea in heart failure patients with preserved ejection fraction poses a particular diagnostic challenge, with obesity (BMI>35 kg/m^2^), age>60 years, treatment with anti-hypertensive drugs, diabetes, and diagnostic indicators from ECG (atrial fibrillations) and echocardiogram as sensitive indicators of its presence (90). In a study of 258 outpatients with chronic heart failure, 9.3% also met the criteria for PD (91). Beyond mere similarity in symptoms, a 2017 review identified PD also as a risk factor for cardiovascular disease, suggesting that PD pathophysiology could initiate or exacerbate cardiovascular disease, although a causal relationship is debatable (92). The shared presentations of cardiac conditions and PD, as well as the high levels of comorbidity, create a diagnostic challenge (see section on chest pain symptom for differentiation of PA symptoms in those with and without cardiac disease).

Panic has also been found to be comorbid with obesity (93); in a sample of 871 obese patients referred for bariatric surgery, 16% presented with comorbid PD (94). With relevance to this review, patients with obesity report an increased prevalence of unexplained dyspnea on exertion (DOE; 95). When assessing differences between obese patients with and without DOE, studies have found no difference in body composition, pulmonary function, cardiorespiratory measures, or oxygen costs of breathing. Instead, obese patients with DOE report increased sensation of the work of breathing over those without DOE, and obese women with DOE report significantly higher anxiety and unpleasantness following exercise than obese women without, which seems to point to a problem of symptom perception or report nature (95).

Given the multidimensionality of dyspnea (96), other less-studied triggers need to be considered. For example, the contraction of respiratory muscles produces respiratory sensations independent of potential changes in PCO_2_ or hyperventilation (97). These muscles are under voluntary control. Their contraction can lead to dynamic hyperinflation, where end-expiratory levels are elevated, and the inspiratory reserve capacity is reduced, limiting exercise capacity and producing dyspnea already in less strenuous physical activities (98). Patients suffering from asthma or COPD are prone to hyperinflation since it can reflexively reduce airway obstruction and relieve dyspnea elicited by it. However, if maintained for longer, this comes at the expense of feeling fatigued and out of breath during regular daily activities. Pilot observations have found that anxious patients with panic symptoms show elevated tension in their chest muscles during the experimental CO_2_ challenge (99).

Functional respiratory disorders comprise a group of conditions that result from respiratory disturbances of unknown origin and are accompanied by dyspnea. Their diagnostic classification has been the subject of ongoing debate (100–102). They are often comorbid with anxiety disorders, in particular PD, or chronic respiratory diseases, in particular asthma, but can also exist in isolation. Exact prevalence rates are difficult to determine because of the uncertain diagnostic criteria and high rates of comorbidity. Most prominently, hyperventilation (103) and vocal cord dysfunction (104) are subsumed under this designation. Hyperventilation itself can be an indicator of a wide range of organic illnesses and can exist without the accompanying anxiety or fear, thus motivating the referral for an in-depth medical work-up. Significant triggers of vocal cord dysfunction have been identified as gastroesophageal reflux disease (GERD) affecting the upper airways/larynx, inhaled air pollutants or irritants, psychological stress, and exercise (105). Although it shares the attack-like onset with PD, dyspnea is often shorter in the range of seconds to a few minutes and felt specifically upon inspiration and in the throat (104). Indicators from flow-volume curves of spirometry and laryngoscopy can help support the diagnosis.

#### Associated characteristics

Psychological processes play a crucial role in the perception of respiratory sensations, including negative emotionality, catastrophic interpretations of symptoms, and the interpretive context of symptoms, associated with the poorer perception of dyspnea regardless of lung function (106, 107). Depressive symptoms have also been associated with greater perceived impairment in patients with asthma, including overperception of airflow obstruction, worse self-reported asthma control, and lower asthma-related quality of life (108). Compared to asthmatics without PD, individuals with comorbid PD and asthma find dyspneic symptoms more distressing during the onset and long-term than those without PD (109). High anxiety sensitivity is associated with more intense feelings of SOB and additional panic symptoms in response to dyspnea induced through inspiratory resistive loads (110), or tensing respiratory muscles by intercostal muscle biofeedback (97). Neuroimaging studies indicate that habituation to dyspnea is influenced by an individual’s level of anxiety in that individuals with low anxiety are more likely to habituate (111). Personality traits such as neuroticism correlate positively with dyspnea intensity (112, 113).

Historically, anxiety disorders have not been included as differential diagnoses in guidelines for dyspnea (114). More recently, published recommendations highlight “functional complaints” as an exclusionary diagnosis after an extensive somatic workup has been completed, which may allude to the functional impairment of anxiety disorders (70). While Wolters Kluwer: UpToDate (https://www.uptodate.com), a widely utilized medical reference tool and clinical decision support resource, includes “hyperventilation and anxiety” as a factor for differential diagnosis, it also recommends that, in the case of patients with respiratory disease, providers “assume exacerbation of their medical disease is cause of the dyspnea until proven otherwise” (115, 116).

### Treatment

No published guidelines are available to discriminate between medically and non-medically relevant dyspnea. Emergency physicians focus on stabilizing breathing and circulation before considering additional diagnoses. Once it has been determined that dyspnea is benign or psychogenic, non-emergent treatment will focus on targeting dyspnea symptoms, including traditional and modified CBT, biofeedback, breathing retraining, physical exercise, and modulation through pharmacological intervention (117–120). Capnometry-assisted respiratory treatment (CART) has been used to target respiratory abnormalities in PD and has demonstrated improvements in respiration and reductions in panic symptoms (121–124) and asthma (125) and comorbid asthma and anxiety (126). CART successfully reduces panicogenic cognitions and dyspnea, which was consistently elicited during the exercises, and appears to have therapeutic effects, possibly by decatastrophizing and reattributing (124, 126). Mindfulness-based interventions, relaxation, meditation, and guided imagery provide evidence for reducing the frequency and severity of dyspnea in patients with lung disease (89, 127). Specifically, a 2019 RCT demonstrated the efficacy of a brief mindful-breathing intervention in reducing dyspnea in patients with lung cancer, COPD, or asthma (127). There is additional evidence that pharmacological intervention and chest wall vibration can modulate dyspnea; however, the utilization of these interventions with comorbid panic is yet to be studied (80, 128).

## Chest pain

### Prevalence and presentation

Chest pain is the second most common reason for adults to present to the emergency department in the United States, accounting for more than 6.5 million visits annually, 4.7% of all visits, with a lifetime prevalence of 20-40% (129, 130). Despite its frequency, chest pain remains a diagnostic challenge (130). Also often non-cardiac, it is the most common symptom of coronary artery disease in men and women. The latter affects over 18 million adults in the US and remains the leading cause of death annually (131). That being said, more than half of patients presenting to the ED with chest pain will have noncardiac origins for their pain, and only 5% will have acute coronary syndrome (132).

Non-cardiac chest pain (NCCP) has an annual prevalence rate of up to 25%, highest in women ages 45-55 or younger than 25 (133, 134). NCCP is also a cardinal symptom of panic, with up to 70% of PAs characterized by chest pain (135). Further, 17%-32% of patients who present to emergency departments with chest pain are found to have PD (136, 137). NCCP is similar to angina pectoris, with many individuals reporting substernal sensations of pressure, squeezing, or burning (133). In the absence of coronary artery abnormalities, chest pain is considered a benign condition with an excellent prognosis (138); however, it often persists and is associated with high disability and cost (139). Studies have found that those with NCCP report lower quality of life, greater psychological distress, and impairment in life functioning, including symptom burden, reduced physical functioning, and excessive medical resource utilization (133, 140). However, chronic NCCP has no demonstrated impact on patient mortality (133, 141).

Causes of NCCP are thought to be both biological (e.g., esophageal mobility disorder) and psychological (e.g., abnormal pain perception, psychiatric disorders, passive pain coping strategies; 142). Fear of cardiac sensations is predictive of NCCP intensity in patients with and without PD; patients with PD and NCCP report greater chest pain than patients with NCCP without PD, and patients with comorbid NCCP and PD report a lower quality of life than patients with NCCP alone (140, 143). However, psychiatric diagnosis is often delayed by years and preceded by costly, often invasive diagnostic testing (144).

### Differential diagnosis

#### Medical event versus panic attack

For those seeking services due to NCCP, the most common biological cause is GERD (145, 146). GERD is diagnosed in up to 60% of NCCP cases and is responsible for seven million annual medical visits (133). GERD occurs when a patient’s stomach content leaks into the esophagus, resulting in pain and discomfort. Additional symptoms of GERD include heartburn, difficulty swallowing, regurgitation of food or sour liquid, vomiting, bad breath, and a sensation of a lump in one’s throat (147). Patients with GERD have a higher incidence of anxiety and PD than the general population, and patients with GERD and anxiety or PD demonstrate more frequent and severe symptoms (147, 148). A diagnosis of GERD is confirmed by endoscopic esophageal mucosal damage and comprehensive screening of symptoms (147). Other biological causes for NCCP include complications within the pulmonary, musculoskeletal, gastrointestinal, and dermatological systems (145, 149).

One cardiovascular condition that shares symptoms with PD but may be difficult to document by ECG after an attack is paroxysmal supraventricular tachycardias (150). ECG assessments after the attack may report only minor abnormalities, but ambulatory monitoring may be used to distinguish this disorder from panic. For those with NCCP, assessing for other panic symptoms that do not usually appear with cardiac symptoms, such as dizziness, chills, hot flashes, and fear of dying, may clarify whether symptoms can be attributed to an anxiety disorder (151). Chest pain does not necessarily indicate greater severity of a panic attack, as it is rated as a less severe symptom than sensations of choking, fear of dying, and paresthesia in the general population that has experienced at least one panic attack (25). It is also rated lower than palpitations, SOB, and dizziness within a treatment-seeking population of panic patients (31).

Importantly, malignant chest pain with a cardiovascular origin, such as microvascular angina, hypertension, and cardiomyopathy, does occur at elevated rates for patients with PD (152). It is unclear whether there is a pathophysiological mechanism by which PD increases the risk for cardiovascular disease. However, frequent activation of stress pathways and chronic inflammation, as well as the higher rates of smoking and hypertension observed in anxiety disorders, have been linked to cardiovascular disease (153, 154). Interestingly, some research suggests that generalized anxiety disorder and not PD may be associated with the onset of cardiovascular disease (155). In patients with coronary artery disease, comorbid PD is associated with an increased risk of major cardiac events, greater disability, higher psychological distress, and lowered quality of life (154). In addition, there is evidence that those with comorbid coronary heart disease and PD have increased mortality, though there is no evidence to support a causal relationship between PD and cardiovascular disease (92, 156).

Acute chest pain, per guidelines put forth on the evaluation and diagnosis of chest pain by the American College of Cardiology/AHA Joint Committee on Clinical Practice Guidelines, should always be evaluated for potential cardiac causes – that is, after history and physical examination, ECG is appropriate to determine whether the nature of the pain is cardiac (i.e. acute coronary syndrome, acute aortic syndrome, pulmonary embolism, acute myopericarditis, valvular heart disease), or attributable to noncardiac causes (130). In cases of stable chest pain (non-acute) with no known history of coronary artery disease, clinical risk assessments may help determine whether patients are low or moderate/high risk for cardiac incidents, which may necessitate stress testing and exercise ECGs (130).

#### Associated characteristics

Severe panic symptoms have been grouped in an overarching factor subtype of cardiorespiratory symptoms, including palpitations, SOB, choking, chest pain, and numbness (31). This subtype also consists of the cognitive symptom of fear of dying, which may be used to help differentiate severe panic symptoms from coronary symptoms (157). Fraenkel et al. (158) examined the predominance of physical (i.e., dyspnea, chest pain) versus cognitive (i.e., intense anxiety, fear of death, and loss of control) symptom reports in patients diagnosed with PD, early-stage ischemic heart disease, and chest pain with a standard angiogram. Cardiac patients predominately reported physical but rarely cognitive symptoms; by contrast, chest pain experiences in panic patients and NCCP patients were dominated by catastrophic thoughts such as fear of death or losing control. In addition, Mourad et al. (4) found that cardiac anxiety was highly prevalent in NCCP, leading to threatening interpretations of the symptoms that result in healthcare-seeking behaviors. Thus, chest pain of unknown origin accompanied by predominant cognitive complaints or greater levels of cardiac anxiety may indicate a lack of cardiac disease (4, 158).

Further, NCCP has been linked to higher rates of psychiatric disorders, such as mood disorders and other anxiety disorders like generalized anxiety disorder, specific phobias, and social anxiety (5, 159). Patients with NCCP and comorbid anxiety or depression symptoms were particularly prone to hypervigilance of cardiorespiratory symptoms (159). This selective focus is due to a misattribution of NCCP symptoms to pathological causes. The prolonged impairment and experience of chest pain following a lack of a cardiac diagnosis may, in part, be explained by continued anxious hypervigilance. NCCP, combined with patients’ attribution to pathological causes, is associated with elevated chest pain and interference with daily activities, even after controlling for anxiety sensitivity and bodily vigilance (160).

Negative angiographies are linked to young age, fear or apprehension before pain onset, pain that does not radiate to the left arm, sharp, not crushing pain, pain located over the heart instead of below the sternum, and inconsistency in reproducing exertion-related chest pain (161). Further, reports have indicated higher cardiac anxiety (4), atypical quality of chest pain (substernal in location, initiated by exertion, and relieved by rest or nitroglycerine) (162), being female (5), and absence of coronary heart disease were also predictors of higher rates of PD in those presenting for chest pain. For instance, Schroeder et al. (163) investigated differences between 240 participants with NCCP versus chest pain of cardiac origin and found that the majority of patients diagnosed with NCCP (70.4%) were more likely younger, female and more sensitive toward somatic sensations than those who had cardiac chest pain (163). Cooke et al. (164) argue that unnecessary angiograms could be avoided by 30% by using the most discriminative pain characteristics (reproducibility, usual duration, and rest) and age (above or below 55). Notably, the authors also recommend assessing the degree of dominating catastrophic cognitions accompanying the experience of pain as they are more indicative of PD (157, 165).

### Treatment

The first-line treatment for NCCP with GERD diagnosis is anti-reflux medication, including proton pump inhibitors and behavioral changes such as diet modifications (166). Reports indicate that most NCCP patients see improvements after receiving this treatment. For those with pain due to esophageal disorders, recommendations may include muscle relaxants, botulinum toxin injection, pain modulators, and surgery (167, 168). Given that psychosocial stressors exacerbate symptoms of GERD, psychogastroenterology has emphasized the benefit of augmenting GI interventions with psychological interventions (169, 170). Trials indicate promising results for psychological interventions, including speech therapy, relaxation, and diaphragmatic breathing (171–173).

When cardiovascular evaluations are negative, patients with cardiac pain accompanied by greater anxiety and cognitive complaints should be referred to mental health providers. Psychotherapy can not only aid in reducing pain but also in treating associated panic symptoms. In 2015, a Cochrane review of 17 randomized controlled trials determined that CBT was beneficial for individuals with NCCP (174). CBT for NCCP aims to decrease distress by restructuring previously catastrophic thoughts, providing education regarding symptom etiology, and exposing patients to feared bodily sensations (175). A 24-week CBT intervention for 113 individuals presenting with chest pain and PD or depression resulted in significant reductions in disease severity, anxiety, and depression (176). In addition to CBT, hypnosis, relaxation training, and guided breathing have demonstrated promise in treating NCCP (167). Pharmacological agents designed to address anxiety and depressive symptoms, such as selective serotonin reuptake inhibitors (SSRIs), are also employed by physicians (167). Viazis and colleagues (177) trialed the SSRI citalopram alongside proton pump inhibitors in 63 NCCP over a 12-week therapy and found complete resolution of chest pain in 68.5% of patients.

## Nausea or abdominal distress

### Prevalence and presentation

Gastrointestinal (GI) symptoms are common in multiple anxiety disorders (178, 179), including PD (180). Nausea or abdominal distress is reported in up to 28% of PD cases (181). Though common, GI symptoms are not reported as being as severe as other subtypes of PD symptoms, namely cardiorespiratory symptoms (25, 31). There is extensive literature on the impact of stress and anxiety on GI conditions and quality of life, particularly when untreated (182, 183). Research on the gut-brain axis has found that higher levels of anxiety may exacerbate symptoms of, both functional GI disorders, such as irritable bowel syndrome (IBS), as well as Inflammatory Bowel Diseases (IBD), and may contribute to maintaining mental health disorders (184–186). Research on the gut-brain axis has shown that there are multiple pathways by which psychological factors can contribute to GI symptoms and disorders (187, 188). Although these mechanisms of etiological determinants have not been fully described, there is evidence for the impact of psychological factors on the developmental course and recurrence of abdominal distress (186, 189).

### Differential diagnosis

#### Medical event versus panic attack

Anxiety is associated with higher rates of GI diseases such as peptic ulcers and Crohn’s disease (190, 191), and twin studies have established underlying genetic factors between GI disorders and PAs (192). Nevertheless, the relationship between psychosocial and abdominal symptoms is most effectively established in Functional Gastrointestinal Disorders, such as IBS (193). IBS is common, accounting for nearly half of all referrals to gastroenterology care, and presents as intense abdominal pain and altered bowel habits (194). IBS is comorbid in PD in 3-17% of PD cases (195). A recent study investigating genetic risk factors of IBS completed a genome-wide analysis of over 50,000 individuals with the disorder. Researchers identified that genes relevant to the susceptibility of IBS were associated with mood and anxiety disorders. Further, researchers found a strong genome-wide overlap of IBS with psychopathological features, such as anxiety, neuroticism, depression, and schizophrenia. Evidence suggests this association is likely due to shared etiological pathways rather than psychopathology causing abdominal symptoms or vice versa (196).

Most patients with IBS (around 70%) present to primary care, while an estimated 25% present to gastroenterologists (197). Only an estimated 5% of all IBS cases present to acute care settings like emergency departments. IBS patients who present to the ED tend to have constant symptoms, psychosocial difficulties, increased healthcare-seeking behavior, more negative illness perception, and more psychiatric diagnoses than IBS patients who present to primary care or gastroenterologists (197). As the most common GI symptoms endorsed during PAs are typically associated with IBS (nausea, bloating, abdominal pain, constipation), and comorbidity between PD and IBS is high, differentiation, particularly during acute distress, might rely on symptom-based criteria known as “alarm features” of IBS (i.e. unexplained weight loss, blood in stool, fever, signs of bowel obstruction, anemia) as described by the American College of Gastroenterology IBS Task Force and Technical Review for safe and timely diagnosis (181, 197, 198). Those experiencing exclusively panic-originated nausea or abdominal distress will likely endorse symptoms peaking within minutes (199). This contrasts with IBS, which is characterized by recurrent abdominal pain and changes in bowel habits, including diarrhea or constipation lasting for hours (198).

Food allergies and intolerances are additional sources of abdominal distress. Seventy percent of the adult world population cannot digest lactose (200). A smaller portion of the population may be intolerant to fat and proteins in lactose-rich products (201). Celiac disease, an immune disease that precludes the digestion of gluten, may account for diarrhea and bloating observed with abdominal distress (202). A food-elimination diet, supervised by a medical professional, may elucidate specific food while maintaining adequate nutrition (203). While symptoms of acute abdominal distress due to food allergies overlap with abdominal discomfort experienced during PAs, allergies produce unique symptoms not seen in PAs, such as swelling, flatulence, diarrhea, vomiting, and abdominal noises. GI symptoms during PAs are typically brief and do not occur exclusively following meals. As such, timing and chronicity of symptoms may help differentiate between disorders, acute abdominal distress attributable to PAs versus food allergies.

#### Associated characteristics

The presence of intense fear may be an essential determinant in differentiating between abdominal distress associated solely with PAs and GI diseases. Those experiencing panic symptoms, including abdominal distress, frequently report fear of losing control or dying (204, 205). Such fears are seldom reported by GI patients, who typically report pragmatic fears such as not having access to a toilet or losing bowel control (206). Those with PA will experience additional physiological symptoms associated with panic, including cardiopulmonary symptoms, sweating, paresthesias, and derealization/depersonalization. PAs as specifiers of other anxiety disorders can be another critical difference from GI diseases. For those with chronic GI, abdominal [symptoms] may arise alongside a stressor/trigger or appear independently (198). If abdominal symptoms occur only in the presence of acute psychosocial stressors, they may be more likely due to panic symptoms and not an underlying GI disorder. However, PA, by definition, presents unexpectedly (2), yet they can be triggered by psychosocial stressors, as seen in social anxiety disorder and PD with agoraphobia (207).

### Treatment

For those who are lactose intolerant, interventions to reduce abdominal distress include utilizing lactose-free foods, ingesting exogenous oral enzymes, and utilizing pre- and probiotics (208), with first-time treatment seekers and those with low impairments being recommended lifestyle changes (209). Recent developments in understanding the pathophysiology of IBS have led to targeted medications for the disorder (209). Additionally, pharmacological agents commonly used to treat PD (tricyclics anti-depressants and selective serotonin reuptake inhibitors) have been shown to help those with IBS address pain and discomfort as a second-line treatment (209). Tricyclics are the recommended agents due to their well-established efficacy and their secondary effect of slowing intestinal transit time through anticholinergic changes.

As there is a reciprocal and reinforcing relationship between anxiety and GI issues, targeting maladaptive thoughts through psychotherapy is beneficial (210). A recent study of patients with celiac disease found that psychological distress and negative illness perceptions strongly predicted and mediated quality of life and GI symptoms (211). Psychotherapeutic interventions for IBS include CBT, relaxation techniques, hypnosis, and psychodynamic therapy (212). CBT and psychodynamic therapies are well-established treatments for PD (213), making them particularly strong choices to target PD and functional gastrointestinal disorders comorbidities (214). Specifically, CBT may target elevated sensitivity and vigilance of visceral sensations through interoceptive exposure (193, 212). Moreover, the enhancement of already prescribed medications (i.e., antidepressants and anxiolytics) with CBT has been beneficial (215). Rapport building between providers and patients is crucial due to the prevalent negative stigma associated with PD and GI disorders (216). A strong therapeutic alliance increases the success rate of referrals for psychological treatment (217).

## Dizziness, unsteadiness, light-headedness, or faintness

### Prevalence and presentation

Dizziness/vertigo is reported in 20% to 56% of the general population (218). Dizziness or vertigo are among the most common complaints in primary care and account for 5.5% of ER presentations (219). Nearly 26 million people in the US visited an ER for dizziness/vertigo between 1995-2004. More than 70% of individuals who complain of dizziness will not see improvement at a two-week follow-up (35, 220). Despite high healthcare utilization, less than 10% of patients undergoing diagnostic procedures for dizziness received a diagnosis (221). Dizziness can have etiological roots in vestibular, cardiovascular, neurological, metabolic, and psychiatric diseases and be benign or indicative of more severe conditions (222, 223).

### Differential diagnosis

#### Medical event versus panic attack

Dizziness may result from hyperventilation (i.e, increased ventilation beyond metabolic demand, 224). In exposure to feared stimuli, deep breaths are associated with dizziness, lightheadedness, and faintness in anxiety disorders such as blood-injection-injury phobia (225, 226). The associated drop in end-tidal PCO_2_ can be expected to constrict cerebral blood vessels, which may at least partially account for additional cognitive and emotional symptoms, such as anxiety and catastrophic cognitions (227). Hyperventilation has been reported in 50-83% of PD cases (6). Various medical, psychological, and social factors contributing to the development and maintenance of hyperventilation should be considered before diagnosis.

Dizziness can also be a result of cerebrovascular or cardiovascular conditions. Indeed, dizziness is the most common symptom in patients with transient ischemic attacks or strokes, particularly those isolated to the cerebellum and brainstem regions (228). Though stroke accounts for just over 3-5% of all dizziness presentations in the emergency department, patients with dizziness have a two-times greater risk of stroke or cardiovascular events than patients without dizziness (229–231). Given this, expedient, accurate diagnoses are paramount. However, non-vascular conditions that mimic acute ischemic stroke in adults, such as psychiatric disorders (including PD) or vestibular dysfunctions, represent 1% to 30% of acute stroke emergency admissions (228, 232, 233). Several studies have suggested using bedside head impulse tests, in which patients are asked to abruptly rotate their heads to identify strokes when dizziness is present (232).

Other abnormalities causing dizziness include orthostatic intolerance, which occurs when blood pressure remains low instead of being restored to adequate levels when a patient is upright, resulting in symptoms of dizziness, blurred vision, and fainting (234). Orthostatic intolerance is common in postural orthostatic tachycardia syndrome (POTS), which is additionally accompanied by a rapid increase in HR within 10 minutes of rising (235). POTS shares several symptoms with PD, including palpitations, dyspnea, chest pain, and sweating. Additionally, scores on panic symptom inventories are similar for patients with POTS and PD (236). However, whether or not patients with POTS are at a higher risk for PD is contested (236, 237). Patients with POTS have several clinical differences that help distinguish it from PD, including known precipitants for symptom onset (dehydration or standing) and worsening symptoms when assuming an upright position (238). POTS is diagnosed by testing to rule out autonomic failure and observation of tachycardia upon posture change (239). Dizziness in POTS is persistent and distinguishable from transient dizziness when a healthy patient stands up quickly. Although anyone can develop POTS, 75-80% are women ages 15-50 years or patients immediately following surgery, trauma, viral illness, or pregnancy (240).

Abnormalities of the vestibular system are the most common causes of dizziness (241, 242). Dizziness can be rooted in peripheral dysfunction in the inner ear, as is the case of ear infections, or in benign paroxysmal positional vertigo. Individuals experience a spinning sensation triggered by changes in head positioning (243). Dizziness can also result from pathologies of the central nervous system, such as concussion, migraine, tumors, and traumatic brain injury (244). There are over 20 vestibular disorders, and most present with the common symptoms of vertigo, dizziness, oscillopsia, and unsteadiness (245). One study assessing vestibular disorders and somatization interaction found that Meniere’s disease and vestibular migraines have the highest psychiatric comorbidity (246). These two conditions are commonly considered differential diagnoses (241). The presence of multiple vestibular symptoms indicates vestibular migraines. In contrast, Meniere’s Disease is a chronic inner ear disorder characterized by severe vertigo attacks, hearing loss, ear fullness or tinnitus, anxiety or fear, diarrhea, and nausea, among other symptoms (247). Finally, hypoglycemia is a cause of dizziness that may resemble PD. Hypoglycemia is a physiological state characterized by a low blood glucose level. It is accompanied by a broad range of symptoms that can include palpitations, anxiety, confusion, irritability, sweating, tremulousness, and in severe cases, coma (248). Though PD and hypoglycemia share common symptoms, effective diagnosis for hypoglycemia is established, and misdiagnosis is rare.

Vestibular dysfunction can be diagnosed by assessing hearing, balance, inner ear functioning, eye movement, and reactions using tone audiometry, rotation tests, vestibular evoked myogenic potentials, and electronystagmography (249). Many of these tests might not be useful in differentiating vestibular dysfunction from PD, given findings that demonstrate vestibular abnormalities exist in individuals with PD (250). Comorbid vestibular abnormality and anxiety or PD are highly prevalent, thus complicating differential diagnosis further (218, 221). It is unclear whether primary vestibular abnormalities cause panic or whether there is a psychogenic vestibular disturbance (251).

#### Associated characteristics

Dizziness is among the most frequently reported symptoms of PAs (31). Patients with dizziness and PD with and without agoraphobia have demonstrated significantly more peripheral vestibular abnormalities than patients with depressive disorders (250). Evidence indicates that individuals with ongoing dizziness have unique personality traits, including obsessive-compulsive traits and labile affect, compared to unaffected controls (252). For instance, in a study of 41 patients, 19 with chronic dizziness and 22 with other vestibular disorders, in which participants answered questions regarding disability, anxiety, depression, and personality traits, individuals with dizziness reported higher anxiety, neuroticism, and openness compared to the healthy controls (253). Previous research with patients endorsing dizziness found that patients had higher levels of fear-avoidance beliefs, depression and anxiety, and perceived disability than healthy controls (254). In a study of 1287 individuals from a representative community sample in Germany, dizziness was reported in 15.8% of the sample, and 28.3% of those with dizziness reported at least one anxiety disorder (such as generalized anxiety disorder, social phobia, or panic) (218). Other studies have shown that 50-85% of PD patients experience dizziness during PAs (255) and some have proposed dizziness as an indicator of a moderate to severe PA (135, 256). Studies have varied concerning intensity ratings of dizziness, showing it on an equal level with the most intense symptoms of palpitation and SOB (31) or rated as less severe than symptoms of choking, fear of dying, and paresthesia (25, 204). However, most assessments, such as the Structured Clinical Interview for DSM-5, evaluate symptoms’ frequency but not the severity.

### Treatment

POTS can be treated with medications such as salt tablets or beta-blockers, selective serotonin reuptake inhibitors (SSRIs), and changes in diet and exercise (235). Depending on the etiology, treatment for vestibular dysfunction may include vestibular rehabilitation, medications, surgery, or lifestyle changes (257, 258). Pharmacological treatments for anxiety disorders, such as SSRIs, show promise in treating psychogenic or psychiatrically comorbid dizziness (258, 259). A case series of nine patients with PD with agoraphobia and chronic dizziness demonstrated a significant decrease in anxiety and the impact of dizziness following three months of imipramine (260). Additionally, SSRIs were seen to have a central balancing effect on vertigo attacks in 12 patients with Meniere’s disease and generalized anxiety disorder (261). However, research in this area is limited to case studies and open-label trials.

Interestingly, one paper discussing the similarities between vestibular dysfunction and PD highlighted the equivalence of vestibular rehabilitation and behavior therapy for anxiety disorders (257). Psychotherapy, namely CBT, has produced improvements in patients with dizziness, and researchers suggest providing psychoeducation about the origin of their symptoms as part of abnormal sensory processing and postural control, which occur in response to a past stressor or vestibular disorder (258). In a randomized control trial, 41 patients with chronic dizziness were assigned to a waitlist or treatment condition consisting of 3 weekly CBT sessions modeled on CBT sessions for PD (262). Treatment yielded significant reductions in dizziness and related physical symptoms, disability, avoidance, and use of safety behaviors. CBT has also been used to augment the efficacy of SSRIs, as demonstrated by an RCT combining CBT and sertraline (263). Ninety-one participants with persistent dizziness were randomized into a control group (sertraline) or an experimental group (sertraline + CBT). Results indicated a significant decrease in dizziness, anxiety, and depression. The experimental group demonstrated significantly lower levels in all three outcomes at weeks 4 and 8 (263).

## Paresthesia

### Prevalence and presentation

Paresthesia describes abnormal sensations, including numbness, tingling, or prickling, typically felt in the extremities, though it can occur anywhere on the body (264). Most individuals have or will experience paresthesia in their lifetime, likely due to benign causes such as sitting, standing, or sleeping in a static position or as a common side effect of medications or treatments such as chemotherapy (29, 265). The distressing experience of paresthesia combined with catastrophic cognitions common in PD can lead to emergency or primary care visits (266). In a study of 616 emergency patients, paresthesia was reported in 61.5% of patients; half (50.5%) had a psychiatric co-morbidity, anxiety/PD (39.2%; 266). Ietsugu et al. (204) found that paresthesia was endorsed by at least a quarter of 1213 respondents with at least one PA in their lifetime. Although one of the less-frequently endorsed symptoms in panic (31), paresthesia holds high discriminative power when present and is one of the better markers of severe PA (25, 204). Paresthesia is also a significant predictor of care-seeking for PA in the ER (8, 204).

### Differential diagnosis

#### Medical event versus panic attack

Paresthesia is commonly reported in peripheral or central nervous system disorders, such as multiple sclerosis, stroke, or orthopedic trauma (267). For many of these conditions, a precise medical diagnosis is evident; however, differential diagnosis is more complicated in some cases. Epilepsy, for example, is often misdiagnosed as, or conflated with, syncope or psychogenic nonepileptic seizures, with reports of misdiagnosis as high as 18%-42% (PNES; 268, 269). Epilepsy is a neurological disorder affecting 1% of the population and is characterized by seizures (270). Research estimates that anxiety disorders are more than twice as likely in patients with epilepsy compared to the general population, and paresthesia is reported in up to 72% of epilepsy cases (26). Due to the similar features and high comorbidity, panic is one of the most prevalent condition that requires differentiation from seizure disorders (271, 272). However, there is research that attempts to delineate the two disorders. A 2014 retrospective analysis of 354 patients with epilepsy and psychogenic nonepileptic spells (PNES) found that patients with PNES reported a statistically higher mean number of symptoms and a greater frequency of panic attack symptoms (273).

Moreover, one-third of epileptic patients experience ictal fear, a sudden unexplained onset of fear at the beginning of a seizure further confounds discernment from panic (274). It has also been posited that PA can result from simple partial seizures with purely mental presentations, including depersonalization, derealization, and affective experiences (e.g., panic, sadness, joy), as evidenced by eventual electroencephalogram (EEG) abnormalities (275). If clinical presentations are indistinguishable, an EEG can identify characteristic abnormalities in frequency bands of electrocortical activity in most cases (274).

Paresthesia is a common and well-recognized symptom of stroke (276). Stroke accounts for about half of all hospital admissions for acute neurological disease and is a leading cause of severe and long-term disability in the US (277), with up to 50% of survivors being chronically disabled (278). As mentioned above, psychiatric disorders, including anxiety or PA, are among the most common conditions mimicking strokes in adults (233). Angiographic studies and exams to evaluate the presence or absence of neurological abnormalities, especially with computed tomography perfusion scans or diffusion-weighted magnetic resonance imaging (MRI), often provide adequate evidence for stroke diagnosis (232). Similar to dizziness, paresthesia may also result from hyperventilation, which has a prevalence of 9.5% in the general population and up to 83% in anxious populations (6, 266).

#### Associated characteristics

Individual differences in interoceptive awareness and higher cognitive processes may play an essential role in experiences of paresthesia (267). Research indicates that the intensity of tingling significantly correlates with body awareness, and attention-related body sensations in paresthesia are more likely the results of top-down cognitive processes (267). As with other somatic symptoms of panic, inadequate or inaccurate interoception may worsen the experience of paresthesia and lead to catastrophizing of benign symptoms; however, interoceptive awareness in paresthesia is less well researched relative to interoception of cardiac or respiratory activity, likely due to the difficulty of obtaining physiological indicators of paresthesias.

There are key characteristics that may aid in distinguishing PAs and epilepsy. PA lasts longer than seizures, with an average seizure duration of a few seconds to about 2 minutes, whereas PA lasts around 10 minutes (279, 280). The average age of onset for PD is 22-23 years old (2), whereas the average age of onset for seizures is bimodal, occurring either before age five or after 65 (281). Additional differences include transient amnesia, motor automatisms, psychosensory symptoms, convulsions, and historical factors, such as a family history of PD or epilepsy (279, 281, 282). Beyond neurological testing, several variables help to differentiate stroke, including atherosclerosis on computed tomographic angiography, atrial fibrillation, older age, high systolic blood pressure, focal weakness, diabetes, and self-report using suitable instruments such as the National Institute of Health Stroke Scale (233, 283).

### Treatment

Treatment for paresthesia may depend on its origin; however, conventional approaches include medications such as gabapentin, lidocaine patches, tramadol, and tricyclic antidepressants for various forms of neuropathy (284, 285). If paresthesia is likely due to PD, psychopharmacological agents for anxiety, such as SSRIs, have shown evidence of reducing the experience of paresthesia (286). Individuals with panic and paresthesia may also benefit from psychotherapy and respiratory biofeedback approaches. CBT, particularly those utilizing interoceptive exposures, are especially advantageous because they focus on addressing fear of bodily sensations and interoceptive awareness. If the paresthesia is a product of hyperventilation, breathing retraining, which uses respiratory biofeedback to reduce respiration abnormalities and hyperventilation, can improve outcomes (121, 122).

## Cultural considerations

It is imperative to understand how panic symptoms present in patient populations from minority cultures to improve diagnosis and treatment. As previously described, panic symptoms often mimic chronic or acute physical diseases. Minority populations in the US are disproportionally vulnerable to chronic medical conditions comorbid with PD and other anxiety disorders. Cardiovascular disease is more common in African Americans than non-Hispanic White people, and asthma prevalence is higher for non-Hispanic Black (13.5%) and Hispanic (7.5%) children compared to non-Hispanic White children (6.4%; 287). Similarly, there are higher incidences and prevalence of diabetes in Hispanic, Indigenous, and African American populations (288, 289). Minority status is often associated with lower socioeconomic status, which increases the risk of being un- or under-insured and unable to manage the growing cost of disease diagnosis and management (241).

Due to specific cultural influences, PD symptoms may also present differently in patients from minority groups. For instance, Hispanics/Latinos present with more significant somatic anxiety within PD, including complaints of heart-pounding, muscular pains or aches, and chest pain or discomfort, than their non-Hispanic counterparts (290). Gazarian et al. (205) noted that Hispanic patients report a greater fear of dying when having PAs. Patients from minority groups are more likely to express symptoms and distress conveyed uniquely in their culture (291). For example, *Ataque de Nervios*, terminology exclusive to Hispanic/Latino populations, is marked primarily by physiological and behavioral symptoms such as trembling, uncontrolled screaming, crying, aggressive or suicidal behavior, in addition to depersonalization or derealization (291). One way to distinguish *Ataque de Nervios* from PD is that it is typically cued by distress, often lasting longer than 10 minutes and associated with fewer symptoms (292).

Additionally, *Ataque de Nervios* has risk factors, treatment history, and symptom endorsement that are different from those of PD (293). Interestingly, non-Hispanic White individuals also report experiencing several of these symptoms, in which case the same differentiating criteria can be used (293). Similarly, *khyâl* attacks are common in Cambodian culture and involve neck soreness, tinnitus, and dizziness, whereas *trung gio* attacks are reported in Vietnamese cultures and include headaches and dizziness (291). Many of these disorders are cultural idioms of distress, including trauma exposure. Thus, in the absence of initial medical causes to symptoms, providers should assess for psychosocial stressors and cultural appropriateness of symptoms to minimize the burden of unnecessary healthcare utilization. Cultural awareness of somatic markers of psychopathology will enable healthcare professionals to educate patients appropriately and incorporate an efficient and effective treatment plan. A randomized control trial investigating CBT plus biofeedback to increase HR variability, culturally adapted for Hispanic/Latino patients with comorbid PD and asthma, found improvements in asthma control and PD severity, with the therapy demonstrating an advantage in improving medication adherence compared to music and relaxation therapy (294). Lastly, research has reported a particularly strong stigma of mental health symptoms reported in minority groups in the US (295). This stigma may cause additional treatment delays for those with panic symptoms. Normalizing these symptoms as a common experience can help reduce the stigma associated with anxiety and panic. Likewise, providers should be prepared to provide referrals to low-cost psychological care; most major metropolitan areas in the US have universities with psychology training clinics that can provide low-cost sliding-scale therapy.

## Conclusion

The inability to accurately distinguish benign somatic symptoms of panic from medical symptoms can create treatment delays and enormous cost burdens. Consequently, resources are needed to assist biobehavioral researchers and healthcare providers in the differential diagnosis of panic-related symptoms. Multiple factors, including high comorbidity and patient and cultural characteristics, reduce patients’ and providers’ ability to distinguish between panic and medical illness symptoms. This review aims to fill this gap by discussing published evidence and current treatments for PD and relevant medical illnesses. Specifically, we reviewed common somatic panic symptoms frequently reported in medical conditions and highlighted criteria for differential diagnosis and available treatment options. We focused on six somatic symptoms (palpitations, shortness of breath, chest pain, nausea, dizziness, and paresthesias) because they are rated as most severe by panic sufferers or most frequently present in medical settings (6, 8, 204). **Table 1** summarizes panic symptoms, their prevalence rates, common medical diagnoses, and considerations for different diagnoses. We found commonalities in the literature, indicating that perceptual and cognitive factors are crucial components in discerning somatic panic symptoms from symptoms caused by medical illness. Specifically, individuals with heightened or inaccurate interoceptive awareness are at higher risk for somatizing and catastrophizing benign symptoms. Patients with poor interoceptive awareness and comorbid psychiatric symptoms are at risk of erroneously labeling benign symptoms as medically relevant. For instance, patients seeking medical consultation for benign palpitations perform worse on heartbeat detection tasks. Patients with medically unexplained symptoms who report higher symptoms are less interoceptive accurate than lower-symptom reporting patients (51, 106). Unfavorable illness perception may also be a predictor of health-seeking behavior. Individuals who perceive their symptoms as signs of a severe condition may be more persistent with medical providers.

**Table 1:** Summary of panic symptoms, their prevalence rates, common medical diagnoses and considerations for different diagnoses.

Symptom	Prevalence in panic	Medical comorbidities	Considerations for differential diagnosis
Palpitations	31%	-Mitral valve prolapse -Arrythmias	Detailed history, physical exam, ECG, Holter monitoring, interoceptive awareness
Shortness of breath/dyspnea	87%	-Asthma -Chronic obstructive pulmonary disease -Obesity -Heart failure -Left ventricular hypertrophy - Functional respiratory disorders (hyperventilation, vocal cord dysfunction)	Detailed history, physical exam, chest film, ECG, hematocrit, pulmonary function tests such as spirometry, reversibility testing with a bronchodilator, methacholine challenge tests to determine airway hyperreactivity, exhaled nitric oxide levels, gas exchange, laryngoscopy
Noncardiac chest pain	70%	- Gastroesophageal reflux disease -Coronary artery disease -Angina pectoris	Detailed history, endoscopy, ECG, stress testing, heart-focused anxiety
Dizziness	16%	-Epilepsy/seizure disorders -Stroke -Orthostatic intolerance /postural orthostatic tachycardia syndrome -Vestibular dysfunction -Meniere’s disease -Hypoglycemia -Benign paroxysmal positional vertigo	Bedside head impulse test, inner ear functioning, rotation tests, and imaging such as CT, MRI
Abdominal Distress	28%	-Crohn’s disease - Functional gastrointestinal disorders (irritable bowel syndrome) -Food allergies	Detailed history, food elimination diets, allergen tests, endoscopy, colonoscopy and biopsy
Paresthesia	25%	-Multiple sclerosis -Stroke -Orthopedic trauma -Epilepsy	Detailed history, EEG, CT, MRI

Our review also serves as a reminder of the necessity of a multidisciplinary approach to symptom evaluation before physical symptoms are classified as evidence of PD. Medical centers and healthcare providers have begun to utilize screeners to identify and flag patients at risk for misdiagnosis, having psychologists embedded in clinics/services with high rates of comorbidity. However, many do not have the resources for comprehensive multidisciplinary care; most often, patients are seen by one provider. This review addresses an urgent need in the reality of healthcare in the United States: though beneficial and gold-standard care model, multidisciplinary healthcare continues to reach only a small fraction of patients. Only a thorough medical work-up will guarantee that symptoms of comorbid conditions are classified correctly and treated adequately.

The extensive differential diagnosis for PD and medical conditions necessitates a detailed patient history, including the time course of the complaint, its severity, associated symptoms, and medical history. Additionally, psychological screeners (e.g., 296, 297) greatly aid the diagnostic work-up and provide additional knowledge on physical symptoms as potential indicators of comorbid or alternative conditions. Beneficial information for healthcare providers are the prevalence rates of PD and relevant medical conditions in their practice, the average age of onset for PD (22 to 23 years), and the average duration of PA (10 minutes) (2). Assessing patients’ life and family history is useful in discerning PD from medical illnesses. For instance, a family history of anxiety or PD and previous PAs may increase the likelihood that symptoms result from a PA rather than a medical condition. Concerning medical treatment, education and reassurance typically do not assuage patient concerns. As described above, psychoeducation and psychotherapy can help address benign symptoms and cognitive catastrophizing contributing to distress. In light of the recent COVID-19 pandemic, symptom differentiation in healthcare-seeking patients is becoming even more critical. Dyspnea and other SARS-CoV-2 infection symptoms overlap with symptoms of panic (298), and fear of COVID-19 has been associated with a tendency to panic (299). Approximately 10-30% of COVID-19 patients suffer long-lasting symptoms, which are recognized as Post-Acute Sequelae of SARS-CoV-2 (PASC), commonly also called “long COVID” (300, 301). Patients suffering from PASC report symptoms months after initial disease onset, including dyspnea, chest pain, persistent cough, and fatigue, among others, with dyspnea and fatigue being cited as the most common (302–304). Thus, recent literature suggests that differentially diagnosing PD and other illnesses will be additionally challenging in the aftermath of the COVID-19 pandemic.

Our review is limited by several factors. Firstly, given the breadth of topics, we opted to provide a resource for biobehavioral researchers and healthcare professionals who study or treat patients with symptoms compatible with PD. In such, this review does not provide an in-depth conceptual analysis of the interplay of symptoms in psychiatric and somatic illnesses (e.g., 6 for conceptual review) or a fully systematic review due to its comprehensive scope. Our review provides the foundation for further analysis and discussion on actionable strategies to improve patient care and outcomes. Secondly, the focus on studies from the United States may have introduced bias and limited the generalizability of our findings to other cultural contexts. The lack of consistency across symptoms in terms of data provided and information covered reflects the inherent variability and gaps within the existing literature, which may have impacted the organization of our review. Finally, medical conditions associated with panic symptoms in this review are by no means exclusive. For instance, individuals with Ehlers-Danlos, a genetic connective-tissue disorder/hypermobility spectrum disorder, are at a relative risk as high as 22.3 for developing anxiety disorders (305, 306). Research supports not only epidemiological evidence, but also extensive clinical and non-clinical association as well as non-human data on the presence of the association between Ehlers-Danlos and Anxiety (307). Efforts are on the way to validate further the newly defined neuroconnective endophenotype clinical construct (and its corresponding instrument) to better capture related somatic and psychological symptoms (308).

Despite these limitations, our review provides valuable insights into the topic and highlights areas for future research and improvement in methodology. Our review discussed literature on medical conditions that can be confused or be comorbid with PD to assist biobehavioral researchers or healthcare professionals in identifying at-risk patients and supporting their proper diagnosis. Although progress has been made in the differential diagnosis of PD and PAs, a considerable need for research on diagnostic procedures remains to aid differentiation between benign versus malignant symptoms. Establishing guidelines to identify patients most likely to misinterpret benign symptoms and direct them to appropriate treatments would reduce costly, invasive testing and prolonged suffering.

## Author contributions

NT: Writing – review & editing, Writing – original draft, Methodology, Formal analysis. SC: Writing – review & editing, Methodology, Investigation, Conceptualization. AR: Writing – review & editing, Writing – original draft, Validation, Methodology, Data curation. JK: Writing – review & editing, Writing – original draft, Validation, Data curation, Conceptualization. TR: Writing – review & editing, Writing – original draft, Validation, Supervision, Conceptualization. AM: Writing – review & editing, Writing – original draft, Validation, Supervision, Methodology, Formal analysis, Conceptualization.
